# Zebra Finch Mates Use Their Forebrain Song System in Unlearned Call Communication

**DOI:** 10.1371/journal.pone.0109334

**Published:** 2014-10-14

**Authors:** Andries Ter Maat, Lisa Trost, Hannes Sagunsky, Susanne Seltmann, Manfred Gahr

**Affiliations:** Max Planck Institute for Ornithology, Eberhard-Gwinner-Straße, Seewiesen, Germany; Utrecht University, Netherlands

## Abstract

Unlearned calls are produced by all birds whereas learned songs are only found in three avian taxa, most notably in songbirds. The neural basis for song learning and production is formed by interconnected song nuclei: the song control system. In addition to song, zebra finches produce large numbers of soft, unlearned calls, among which “stack” calls are uttered frequently. To determine unequivocally the calls produced by each member of a group, we mounted miniature wireless microphones on each zebra finch. We find that group living paired males and females communicate using bilateral stack calling. To investigate the role of the song control system in call-based male female communication, we recorded the electrical activity in a premotor nucleus of the song control system in freely behaving male birds. The unique combination of acoustic monitoring together with wireless brain recording of individual zebra finches in groups shows that the neuronal activity of the song system correlates with the production of unlearned stack calls. The results suggest that the song system evolved from a brain circuit controlling simple unlearned calls to a system capable of producing acoustically rich, learned vocalizations.

## Introduction

Songbirds, which make up about half of all extant bird species, have the ability to learn complex vocalizations like song and certain types of distance calls beside their innate call repertoire, whereas the closely related suboscine species produce only unlearned song and calls. The emergence of the ability to produce learned vocalizations is associated with the evolution of the forebrain vocal control system, an interconnected network of brain nuclei that shapes the song during learning and organizes the motor output when singing [1], [2]. At best, only rudimentary traces of this system are found in the non-learning relatives of songbirds [3]. Therefore, the vocal control system is thought to be uniquely devoted to the control of learned sounds. The evolutionary steps that led to the development of the learning-related forebrain vocal system are as yet unknown, but it seems reasonable to assume that the song system has evolved from circuits driving simpler unlearned vocalizations.

**Figure 1:**
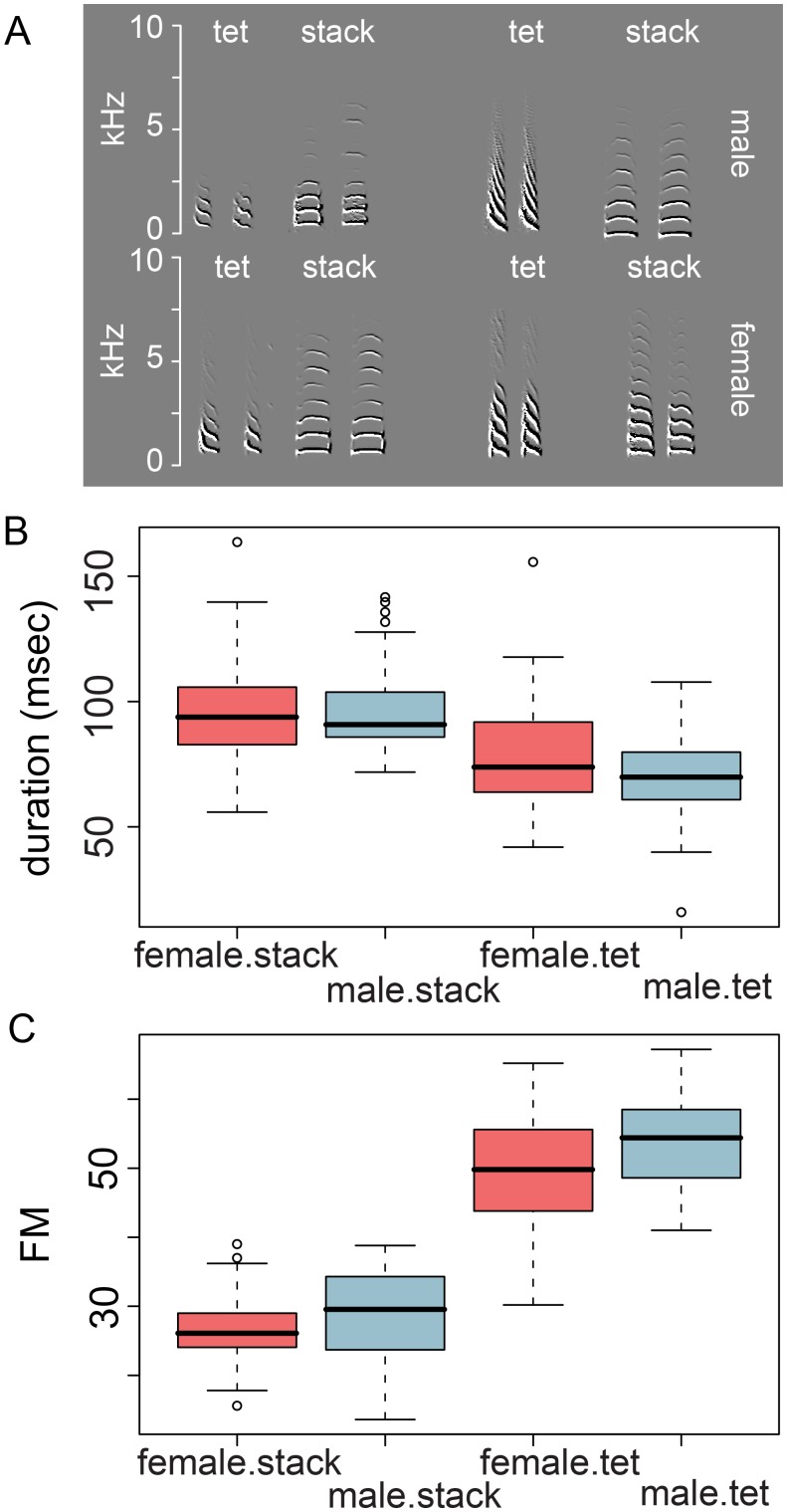
Duration and FM of tet and stack calls in 6 pairs (10 randomly selected measurements per animal). **A.** Examples of tets and stacks. Clearly, tets are much stronger frequency-modulated than stacks. Note that our wireless microphones show more power in the lower frequencies as compared with external microphones since they record the near field. **B.** Tet calls had shorter durations than stacks (P = 1.90e–19). In females, duration was slightly longer than in males (P = 0.0025). **C.** Tets had higher FM-scores than stacks (P = 0.0001) whereas FM-scores were generally lowest in females (P = 0.0003). All tests: REML in JMP10 with pairs as random factor. Pitch was not different between tets and stacks (not shown).

In contrast to learned vocalizations, all birds, including songbirds such as the zebra finch, produce an array of unlearned call types that is present in both sexes [4], [5]. Zebra finches use soft “tet” and “stack” calls (Figure 1A), which are not learned and are thought to be important in close range communication [6]. They are produced in very large numbers [7] by both males and females [8]. Although these calls are not learned, learning might be required for their timed initiation. The evidence for the precise role of soft calls in communication is, however, anecdotical. Calling exchanges in a social setting can only be determined when the calls can be unequivocally ascribed to each individual. Therefore, we have used miniature wireless microphones carried by the animals to study the patterning of vocal interactions within pairs as well as in groups of zebra finches. In this paper we identify mutual stack calling as a defining property of the pair bond.

Since, like stack calls, song is used in a social context, the association of the song control system with communicative calling activity might shed light on its evolutionary history. The premotor nucleus RA (nucleus robustus arcopallialis) is part of the song motor pathway [1], [9]. RA is electrically active during the production of learned vocalizations [10], [11] and controls the spectral and temporal properties of song elements of zebra finches [12]. RA, therefore, is a logical starting point for associating brain activity with stack calling exchanges. Moreover earlier studies suggest an involvement of RA in unlearned call production [12], [13]. Therefore, we studied brain and auditory activity in small groups of socially interacting zebra finches, each animal carrying a wireless microphone. Each male had electrodes implanted into RA to record neuronal signals during vocal production while moving freely. In this way we show that neurons in the song control system perform precise and pronounced burst firing prior to stack calling. Thus, RA has a function in the control of unlearned vocal social interactions. Based on this, we propose an evolutionary scenario in which the song control system evolved from a system that controlled unlearned sounds that were used to communicate with particular conspecifics in a social group; a process that involves learned sensory-motor integration.

## Results

### Associated stack call production between partners

Single pairs of zebra finches (N = 35 pairs) carrying wireless microphones were kept in sound-proofed boxes for at least 7 days. Each pair produced several thousand soft, short calls in addition to contact calls and male song per day (Figure S1). Although not every pair produced tet calls, we compared tet and stack calls in order to be able to obtain a reliable criterion to identify stack calls. Tet calls were shorter than stacks (Figure 1B; P = 1.9e–19), and both types of call were shorter in males than in females (P = 0.0025; call type × gender interaction: P = 0.095). Stack and tet calling rates were not significantly different between males and females (10 pairs, paired t-test; stacks: P = .86; tets: P = .56). Stack calls are easily distinguished from tet calls on the basis of a large difference in FM (Frequency modulation; Figure 1A, C; call type: P<0.0001). FM was larger in males than in females (gender: P = 0.0003; interaction: P = 0.3267). In the following we will focus on stack calls since these were produced reliably at high rates.

**Figure 2:**
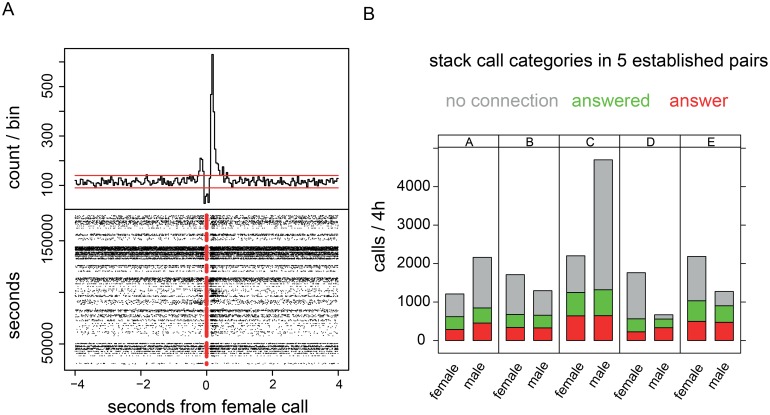
The detailed relationships between stack calls produced by pairs of zebra finches. **A.** The temporal relationship is shown as a peristimulus-time-histogram (PSTH; upper graph), where the onset times of the male calls are aligned to the onset times of the female calls. The horizontal lines in the PSTH are the 1% confidence intervals. In the raster diagram (lower panel) the female calls are shown as red dots. Each male call is represented by a black dot. The probability of male stack calls occurring within half a second before or after the female stack is clearly and significantly increased. Data from one pair kept in a soundbox: 8472 male stacks and 11047 female stacks were recorded in a 35 h period (5 days with 7 h of recording each, starting at 8∶00 AM). Binwidth is 100 msec. **B.** Relative contributions of calls that were answered, were an answer, or were not connected to any stack call of the partner. Data from 5 established pairs.

The production of the stack calls by two partners was clearly time-locked, indicating that many calls were produced in response to the calling of the partner (Figure 2A). Out of the 35 pairs recorded, only two pairs did not show clear correspondences between stacks. The number of stacks that were themselves an answer, and the number that was answered showed less variation than the total numbers produced: in each of 5 pairs that we analyzed in detail and had developed a symmetrical calling relationship the number of calls produced varied 7-fold, whereas the number of stacks that were either an answer or received an answer varied between 1320 and 558, slightly more than twofold (Figure 2B).

### Properties of answered and unanswered stacks

Since not every stack call produced an answer in the partner, we parsed the stack vocalizations into those that were answered, the answers and unconnected stacks. Stacks that followed the partner’s stack within 0.5 sec were labeled “answer”, those followed by a stack call of the partner were “answered”, and stacks falling outside these two categories were called “no connection”. The fundamental frequency, wiener entropy and duration of these calls were determined for stretches of 4 h in 5 pairs. Differences of acoustical components between birds showed up as interactions in the analysis, but there was no overall consistent feature that distinguished between answers, answered and unconnected calls (Figure S2). We have also looked into second order categories (e.g. calls that are an answer and are in turn followed by a stack of the partner). This did not yield any clear differences. The finding is representative of all pairs with clear calling relationships.

### Call patterns in social groups

**Figure 3:**
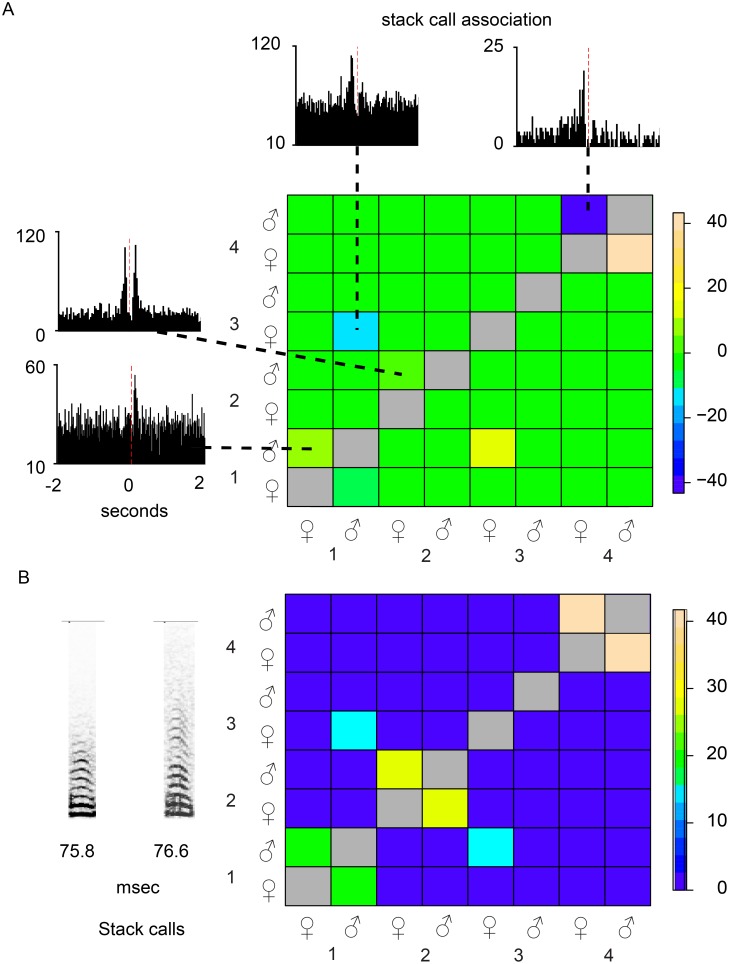
Charting calling relationships in a group. Four pairs of zebra finches. Based on the PSTH, metrics were calculated to describe the strength of the correlation between stack calls. The calls were recorded with carry-on microphones. Indicated in the upper matrix (**A**) are the PSTH’s from which the metrics were calculated. The animals to whose calls the PSTH’s were aligned are indicated along the left vertical side of the matrix. The animals whose calls were counted in the histograms are indicated along the bottom of the matrix. The lower matrix (**B**) shows the strength of the relationship regardless of symmetry. The matrix shows different possible calling relationships. For instance, pair 4 and pair 2 do not call with any other animal in the aviary. In contrast, the male of pair 1 is answered not only by the female of pair 1, but also by the female of pair 3. The partners of pair 3 are the only pair that did not interact at all in our experiments with groups. The associations of the callers with themselves are grayed out because they represent autocorrelations, whereas all the others are cross-correlations. Two sonograms of stack calls are shown in panel B. Duration in msec is shown underneath the sonograms.

We used a new cohort of zebra finches to determine the patterns of interactions mediated by stack calls in group-housed birds. Three groups of four, three groups of three pairs and five groups of two pairs were kept in aviaries and each individual was equipped with a backpack microphone. Prior to the group housing, pairs were kept in soundproofed boxes and after two weeks as a pair, they typically had established a pattern of stack calls that showed significant association. After the group had settled for at least one day, a matrix of association indices was calculated based on the simultaneous wireless microphone recordings of all individuals in the aviary (Figure 3). Pairs that did not establish a calling relationship during the initial week also did not show mutual calling during group housing (e.g. pair 3 in Figure 3). The calling associations persisted unchanged when the pairs were again separately housed in sound boxes. Mutual stack calling, therefore, is likely to define pair bonding. Although mutual stack calling mainly occurs between bonded partners, we occasionally recorded exchanges of stack calls between animals in different pairs. This is also illustrated in Figure 3 where a calling exchange exists between the male of pair 1 and the female of pair 3. Figure S7–9 provide a summary of all the experiments with group-housed zebra finches.

### RA neuron firing is associated with call production

**Figure 4:**
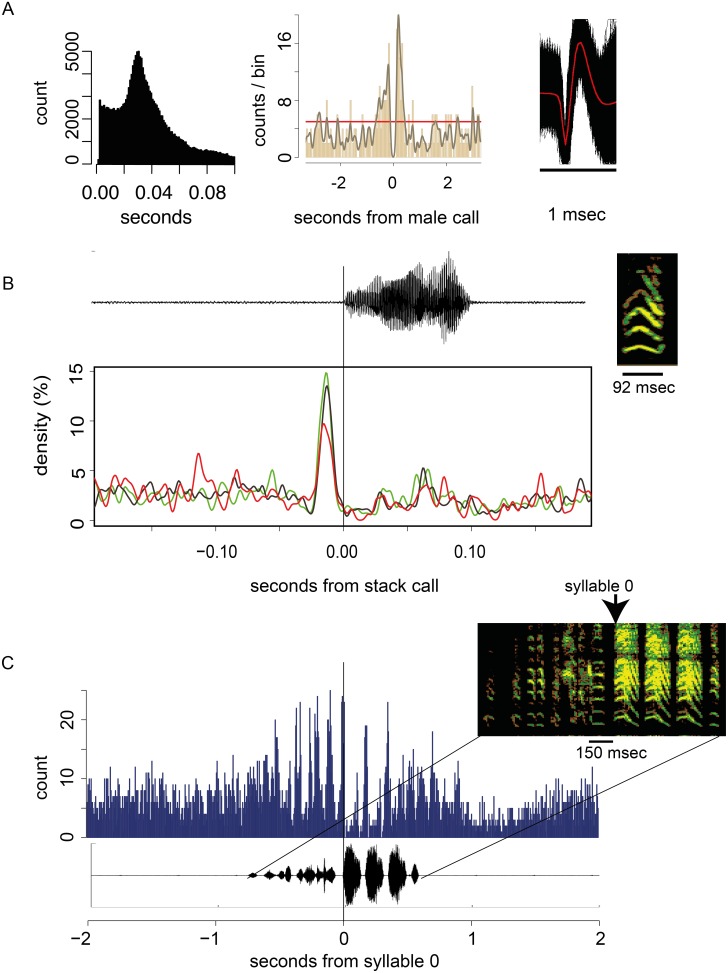
Activity of RA neurons associated with calling and singing. All data from one representative example where the same RA unit fired during song as well as before stack call production. The recording was 4 h. **A.** Properties of the recorded unit and the stack calling exchange of the recorded male with his partner. Left: Interspike interval (ISI) histogram of the unit that was isolated after sorting. The histogram describes a neuron that has a modal ISI of 30.4 msec, which is typical for RA neurons recorded in free moving finches. Center: PSTH of male and female stacks aligned on the 137 male calls. Right: 196642 superimposed waveforms of the unit. **B.** RA activity associated with different stack call categories. RA unit firings aligned to the onset of the stack call. The call is amplified x10 as compared with the song shown in C. Stack calls categorized as “answered” (green, N = 44), “answer” (red, N = 28), or “no connection” (dark grey, N = 65) are associated with elevated RA firing before the call is produced. The RA activity patterns are very similar and seem independent of the stack call category. The call has an average FM value of 24.3 which is well within the range for stacks. The stack does not resemble any song syllable **C.** Binned activity of an RA neuron, aligned to 33 songs produced by this animal. The pattern is aligned to the first of the three repeated syllables (arrow). Binwidth: 5 msec. During song production, the firing rate of the unit corresponds with specific syllables.

In 20 pairs that were kept in soundboxes and recorded with a central microphone, the males carried a chronically implanted tungsten electrode connected to a transmitting high-impedance amplifier [14] to record electrical activity in RA while free moving. In all cases we could differentiate between two different stack calls that were, in 17 cases exchanged between partners (Figure S10). One of these stacks was invariably associated with RA activity (Figure 4; Figure S10). This stack call was in all probability the call of the male. Moreover, in 5 experiments where the stack calls could be attributed unequivocally to each of the individuals through the use of backpack microphones, the male call was always associated with RA activity (Figure S11).

The multiunit recordings were sorted and based on waveform and ISI (interspike interval) histogram putative single units were identified (Figure S10, 11). The RA units in these free moving animals had modal ISI’s of 38.04±9.83 msec (N = 26). Under these experimentally challenging conditions, firing was significantly modulated (exceeding 1% confidence limits calculated from 1000 randomizations of call times) during stack calls in 22 out of the 25 males (Figure 4). In 17 males, firing was increased significantly during singing, and in 16 of those, RA modulation occurred during stack calling (Table 1).

**Table 1:** Association between neuronal activation, stack calls and song in 26 units in 25 pairs.

		song			
		associated	no association	not present	total
	associated	16	0	7	23
stack	no association	1	0	2	3
	not present	0	0	0	0
	total	17	0	9	26

Out of 17 units where both stacks and song were present in sufficient quantity to permit statistical analysis, 16 had firing patterns that were associated both with stacks and song.

The associated firing patterns in RA in 26 units from 25 birds can be classified as follows: Excitation before and during the call occurred in 15 cases, there were 6 instances of inhibition, a biphasic response (inhibition followed by excitation) occurred in 2 cases. Three units showed no significant response. Figures S10 and S11 summarize the results for all RA recordings. We have observed no instance where a stack call was incorporated into a song motif, even though many songs contain syllables that have a stacked sonogram.

In sum, these results show not only that RA firing is associated with stack calling, but also that the same units may be involved in the production of both the unlearned stack call and the learned song.

## Discussion

All birds produce calls for communication [15]. Loud alarm and so-called “long” (or “distance”) calls are often produced anti-phonically in a variety of mammalian and bird species [16]–[19]. We here demonstrate that vocal communication takes place between male and female zebra finches using soft stack calls. Stack call exchanges occur primarily within bonded pairs, suggesting that the unlearned stacks are important in confirming the pair bond, similar to behaviors like clumping and allopreening [20], [21]. The selective responsiveness to the partner’s stack calls also strongly suggests that zebra finches can distinguish between the calls of different individuals in the group. In the case of the loud learned contact calls used for long distance communication, it has been shown that zebra finches do recognize their own partner [18]. Bilateral communication patterns at the nest have been shown to comprise of different soft call types, all designated as “tet” calls [22].

Since pair-bonded zebra finches live in larger social groups the observed specific call exchanges among group members require to learn the individual call signature of other group members and to respond in a timed fashion to specific calls. Such timed responses are not an automatism since we find that not all calls of the mate are answered or are answers. Short-range contact calling could help the animals to locate their partners in a flock. Why not all stack calls of the mate are answered despite being uttered within hearing range of the call receiver remains unknown.

RA is part of the so-called song control system and organizes the motor output of this system. This nucleus is critical for the production of song as well as learned aspects of distance calls in male zebra finches [2], [11]. We find that RA neurons are also active preceding stack calls. We, therefore, speculate that the song system plays a role in call-based communication between bonded partners. This implies that partners are able to recognize each others’ calls. Since zebra finches produce several thousand stack calls per day (Figure 2B) call-based social communication seems to be a major function of RA, next to song control.

The fact that RA is controlling innate calls as well as learned vocalizations allows speculation about the evolutionary origin of the song control system. Since vocal learning occurs only in three not closely related avian taxa (songbirds, hummingbirds, parrots) and since the closest relatives of the songbirds, the sub-oscine passerines do not show vocal motor learning [23], it is parsimonious to assume that production of innate sounds is the evolutionary older situation.

RA is most clearly defined both morphologically and neurophysiologically in oscine songbirds. In suboscines areas analogous to RA have been described [3], and show concentrations of RA-like cells, but this cell group is not nearly as clearly delineated as in songbirds. During songbird evolution, this RA-precursor could have extended its role from the control of innate calls to learned songs. Our finding that many RA neurons fire milliseconds before innate calls are produced, supports this hypothesis. In particular, since stack call controlling neurons are also involved in the production of learned song syllables, RA is not composed of two separate sub-circuits dedicated to either learned or innate sounds but the same neurons do both, i.e. carry out an evolutionary basic and a derived task. Further, the RA firing patterns suggest an involvement not only in calling per se but also in precisely timed call exchanges between partners, which requires learning.

The symmetry of the call exchange between males and females is a further reason why RA and possibly the rest of the song system might have evolved first as a brain area to control the exchange of innate vocalizations. The song system is present in males and females of all songbirds, even in species with non-singing females such as the zebra finch; song areas such as RA are only composed of less and smaller neurons in non-singing females that are nevertheless functionally connected with the syringeal motor neurons [24], [25]. Further, singing in females occurs in very many songbird families [26]–[28]. Together these data suggest that the differentiation of singing and the song system in both sexes is the ancestral situation. Thus, the ancestral function of a RA-precursor should be in the control of a vocal behavior that occurs in both sexes, such as call exchange between males and females.

The study of brain activity in awake birds has contributed greatly to our understanding of bird song learning and production [10], [29], [30]. Until now, recording in moving animals imposed restrictions on the freedom of movement, because the animals were tethered, which made it impossible to study neuronal activity in social groups. With our lightweight radio transmitters we make available a method that allows us to record the signal of deep brain electrodes and individual vocalizations synchronously. This enables us to relate individual signaling behavior with the underlying neuronal pattern in a group of zebra finches living in an aviary which provides insight into the evolutionary link between innate call production and learned song.

## Methods

### Ethics statement

Both transmitter types, the surgical procedure to implant the deep electrodes and bird maintenance in sound boxes and aviaries were approved by the government of Upper Bavaria, “Sachgebiet 54 – Verbraucherschutz, Veterinärwesen, 80538 München” with the record number: Az. 55.2-1-54-231-25-09. All further animal husbandry or handling was conducted according to the directives 2010/63/EU of the European parliament and of the council of 22 September 2010 on the protection of animals used for scientific purposes.

### Animals

Experimental birds were adult male and female zebra finches (*Taeniopygia guttata*) obtained from our breeding facility. In the experiments with single pairs the birds were kept in wooden cages, placed in custom-made, soundproofed boxes. The equipment of each box comprised a microphone (type C2, Behringer, Willich-Münchheide II, Germany), and a telescopic antenna for wireless transmission.

We kept the zebra finches in a 14/10 Light/Dark cycle (fluorescent lamps), 24°C and 60–70% humidity. The experiments with social groups were performed in 2×2×2 m aviaries that had a perspex roof, and were equipped with branches, plastic trees and perches. Crossed-yagi antennae were mounted over the aviary.

We used 74 animals (37 males, 37 females) in the behavioral experiments. Six pairs were only observed when kept as pairs in soundboxes. 31 pairs were observed in groups (Figure S7–9). Two males and two females also performed in an electrophysiological experiment (TG4 and TG12; Figure. S7, S11). 25 males carried implanted electrodes for neuronal recordings. Each of these males was accompanied by a female, 5 of which carried a wireless microphone. One of the males also carried a wireless microphone. The total number of animals adds up to 120.

### Wireless sound recording

Wireless microphones, weighing 0.6 g, including the battery, were developed in-house (Microphones: Knowles Electronics, FG23329; Figure S3A). Silicon tubing was attached to the microphone and two loops were formed, one around the neck, and one around the base of the tail taking care to place it rostral of the cloacal area. Behavioral effects of this backpack occur during the first 24 hours after mounting the microphone. After one day of adaptation the birds showed more self preening activity but apart from that seemed to be unaffected in moving and behavior (Movies S1–S3). The microphone faced towards the body to enhance the specificity of the recording (Figure S4, Movie S4). Crossed yagi antennae were used (Winkler Spezialantennen, Kreuzdipol 300, directional antenna for 300 MHz, clockwise). The frequency modulated radio signals were received using AOR5000 communication receivers (AOR, Ltd., Japan) with the audio bandwidth set at 12 KHz (–3dB). The signal was decoded as FM with intermediate frequency bandwidth set at 110 KHz. In addition we used AOR8600 receivers that were modified to have an audio bandwidth of 12 kHz. Signals were either fed into an 8 channel audio A/D converter (M-Audio 1010; 22050 Hz) and recorded using custom written software, or registered on a DASH8X data recorder (Astro-Med, Inc., RI, USA) at 25 KHz. Analysis was based on continuous recordings of all channels.

### Sorting vocalizations

In order to analyze the temporal relationships between the different vocalizations and their associated neuronal activity, the sounds produced by the animals were classified and time-stamped using segmentation followed by sorting. The sounds registered by the wireless microphones were transmitted continuously. The received audio signals were written to WAVE files that were stored on hard disk. Each animal was recorded at least 4 h per day during an average of 4 days [31]. From these sound files, sounds were extracted using a trigger level set by the user. The sounds were converted into sonograms assembled from 256 point fast Fourier transforms (Intel libraries). This procedure produced a large number of sonograms each describing a syllable, a call, or any other supra-threshold sound. From the sounds the average frequency, modal frequency, fundamental frequency (first peak), Wiener entropy, duration, and their standard deviations were calculated and the sounds were subsequently clustered. The experimenter was free to select which of the above features to use for clustering. Analysis was done using custom software written in Delphi Pascal for Windows and C++ on Apple Macintosh. Sorting was done using a k-means clustering algorithm (Hartigan, 1975) starting with two clusters and splitting new clusters off, one at a time. After clustering, we removed clusters that were not vocalizations as can easily be concluded from inspection of the sonogram. In addition, every cluster was viewed and mistakes were corrected based on visual inspection. The result was stored as bitmap pictures of all the vocalizations in each cluster (Figure S5). Additional acoustic features were extracted using Sound Analysis Pro software [31].

Normally, since the calls are very soft, there was no discernable sound visible that could be attributed to other animals in the aviary or soundbox. A further check of inadvertently recorded vocalizations from an animal other than the focal individual is provided by the fact that in such a case the vocalizations occurred simultaneously in different channels, which was easily determined. However this occurred rarely. Further, the frequency content of the backpack microphone recording was biased to low frequencies, whereas external signal leaks were characterized by a lack of these.

### Analysis of vocalization patterns

After sorting of syllables and calls, their onset times were used to determine the temporal association between the vocalizations of the different animals, both when kept in pairs and in groups. Cross-correlation was determined using peristimulus time histograms [32] (PSTH). Records of the onset times of the different vocalizations were used to construct the histograms where the occurrences of calls (and syllables) of one animal were aligned to specific vocalizations of another animal. Confidence limits were constructed using 1000 runs with the source vocalization placed at random times in stationary epochs of the recording. The strengths of the calling associations were quantified by calculating a metric as follows:

Response strength calculation is based on a PSTH consisting of 2×80 bins of 50 msec. General response strength:
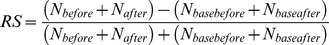
where N_before_ and N_after_ are the counts in the 9 bins before and after the start of the source event ( = call) and N_basebefore_ and N_baseafter_ are the first and last 9 bins in the PSTH. Directionality is calculated as follows:
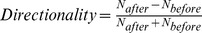



The above index was calculated for each combination of vocalizations and this matrix was further analyzed in R [33]. PSTHs’ that had less than 160 occurrences overall ( = less than 1 per bin on average) were not used to calculate an index. Pearson’s Chi-squared test for goodness of fit was used to determine whether the interaction was significant at p<0.05. We tested the hypothesis that the counts in the four periods used to calculate response strength did not differ between periods. Only when the counts were significantly different the response strengths were used in the matrix.

When an index was not accepted, it was set to missing in the matrix, and for plotting purposes it was set to zero.

### Chronic recording of neuronal activity

To record the electrical activity of RA neurons in free moving animals we have developed a lightweight (1.0 g) telemetry device that wirelessly transmits (multi)unit brain activity and that has no effect on locomotion and vocal activity two days after implantation (Schregardus et al., 2006; Figure S3B). The transmitters used in the current study are a further development of the device with longer battery life (∼7 days), more frequency stability and a longer range at the same weight. Regular telescopic whip (Nagoya Antenna, Taiwan) or tuned crossed yagi antennae (see above) were used, that were connected to AOR 5000 or modified (see above) AOR 8600 receivers.

Each event that was above threshold was captured by peak detection and written into a 64 byte record as reported earlier (Jansen and Ter Maat, 1992). Waveforms were then sorted using a k-means sorting algorithm and further analyzed using custom software.

### Implantation of deep electrodes

The birds were anesthetized using isoflurane inhalation (0.8–1.8% at 0.5l O2/min). The birds were kept warm using a heating pad and wrapped in a thin gauze blanket. The skin of the head was plucked, disinfected and treated with a lidocain (Xylocain Gel 2%, AstraZeneca) containing cream. After a window was opened over the bifurcation of the midsagittal sinus which served as reference, a second window was then made over RA and the dura was opened. A 2 MΩ tungsten electrode (FHC, Bowdoin, USA) was then lowered into RA using a Luigs and Neumann SM-5-remote control system manipulator. The reference electrode was a platinum wire (0,025 mm, Goodfellow) inserted between skull and dura mater. The connectors of reference and recording electrodes were fixed in place using dental cement (Tetric evoflow refill, Ivoclar Vivadent). The connectors serve as a support for the transmitter (Figure S6).

During insertion of the electrode, electrical activity was amplified using a DAM 80 (WPI, AC Differential Amplifier) amplifier, and monitored online using a continuous update of the ISI of Schmitt-triggered spikes. RA activity of projecting neurons was relatively easily recognized by the typical ISI histograms of the spikes [34]. In an initial series of experiments, the location of the electrode was determined using electrolytic lesions. A lesion was made at the recording site and every 500 µm when retracting the electrode. There was a one-for one relationship between finding the RA-typical ISI and the location of the lesion in RA. With 6 implanted males a lesion was made at the end of the experiment. In all 6 cases, RA contained the lesion and the recordings contained a unit that had the interspike interval distribution that is typical for RA projection neurons [35].

### Statistics

Analysis of acoustic parameters was carried out in JMP10 (SAS Institute Inc, Cary NC, USA). Restricted maximum likelihood (REML) with pair ID as a random factor, gender and call type as fixed factors was used to compare acoustical parameters of calls recorded from pairs in sound boxes. All other analyses were done in JMP10 or R [33].

## Supporting Information

Figure S1 **Comparison of the intensities of song, contact call and soft call.** Recording from two pairs in a 1×1×1 m aviary using a central microphone with an all-round sensitivity pattern. Stretches of recording containing representative exemplars the various vocalizations were pasted together to illustrate the relative sound pressure (dBFS, dB Full Scale) of the different vocalizations. The soft call selection contains three tet calls (red dots). The songs are from each of the two males.
(EPS)

Figure S2 **Acoustical properties of answered an unanswered stack calls.** For each of 5 pairs pitch, entropy and duration were measured of all stack calls that were produced in a 4 h period. These calls were subdivided into three categories: answer, answered and no connection. Means and standard deviations are shown. Each pair is shown in a different color. No consistent trends are present in any of the three features. Together, these results do not suggest that the three categories have different acoustical properties.
(EPS)

Figure S3 **Transmitters used in this study.** A. Wireless microphone. The device weighs 0.6 g, including the battery (size 10, pr70 hearing aid battery). Battery life is 12–14 days transmitting continuously. Range is 5 m. We operate the device without an external antenna in order to minimize interference with behavior. B. The electrophysiology transmitter weighs 0.91 g including two batteries (size 10, pr70). Battery life: 8 days. Range is 10 m. The examples in this figure have no batteries inserted. The coin is a 1 eurocent coin, diameter: 16.25 mm. C. Circuit diagram of the microphone transmitter. For more information about the circuit diagram and printed circuit board layout please contact the corresponding author.
(EPS)

Figure S4 **Selectivity of microphone transmitter recordings.** The wireless microphones were mounted with the microphone facing towards the animal’s body. The outward facing parts of the transmitter were covered either in cloth or shrink tubing. A. The top panel shows an experiment with 3 pairs in an aviary measuring 1×1×1 m The wireless (individual) microphone compares with a general microphone mounted in the aviary. Whereas the general microphone records all calls and other sounds made by the six animals, the wireless microphone mounted on an individual selectively records one bird’s vocalizations. Note how the second (tet) call is obscured in the general recording by the call of another individual (red arrows). B. The bottom panel shows a pair of clippings from a 6 channel recording. Sonograms are shown of both channels. Clearly, the vocalizations of the partner animal can be separated from the animal’s own vocalizations by setting a reasonable threshold, as well as by viewing the frequency content of the sonograms. Low frequencies are predominant in the loudest sonograms, probably because the microphones operate in the near field, whereas the crosstalk is characterized by the virtual absence of low frequencies.
(EPS)

Figure S5 **Accuracy of syllable sorting.** Example of a song syllable sorted on the basis of mean, modal and fundamental frequencies as well as their standard deviations, duration and wiener entropy and its SD.
(TIF)

Figure S6 **Surgery details.** During fixation of the connectors in the skull surface and the subsequent disconnection of the electrodes from the input probe of the amplifier used during implantation, it proved essential to prevent any kind of mechanical stress on the electrodes. Although the battery life of the device normally lasted longer than the experiment, sometimes the batteries had to be exchanged. This involved removing the transmitter from the implanted connectors, which could cause the electrode to dislodge. To stabilize the construction, a pin was cemented in with the electrode connectors. Holding this pin with small pliers prevented movement of the electrodes and stress on the skull when the transmitter was plugged in or removed.
(EPS)

Figure S7 **Association matrices in five groups of 2 pairs show significant interactions between males and females.** The males and females are arranged according to previous pairing in a soundbox. Pairwise interactions between males or between females did not occur in our experiments. As an example, TG4 has one pair that engages in mutual calling (response strength * 100 is color coded), whereas the male of the other pair answers to the calls of the female of the pair mentioned previously as shown by the yellow color in the Directionality matrix.
(EPS)

Figure S8 **Association matrices in three groups of 3 pairs.** To clarify the absence of calling among males as well as among females, the males and the females are shown grouped together for experiment AG2 in the small matrices.
(EPS)

Figure S9 **Association matrices in three groups of 4 pairs.** The small matrices under AG3 again show how vocal stack exchanges are limited to contacts between the sexes.
(EPS)

Figure S10 **Recordings from RA-implanted males.** A central microphone recorded vocalizations. The RA recordings are arranged according to type of response. Significance was assessed by randomizing times of occurrence in the relevant sections of the recording and calculating the PSTH, and repeating this 1000 times. Lower and upper limits were determined by the lower and upper 5% of the counts for each bin. The response was considered significant when the count was consistently outside these limits for at least 10 msec. Absence of data indicates that there were too few occurrences to produce a meaningful PSTH.
(EPS)

Figure S11 **Recordings where calls can be unequivocally attributed to individuals.** There were two ways in which this was achieved. 1.) Recordings from RA-implanted males. A central microphone recorded all vocalizations. The female carried a backpack microphone. In this way, female vocalizations in the general microphone recording were identified. The other stack calls were then ascribed to the male. In this case the stack calls were associated with altered RA firing in one male, no RA-modulation in the other. 2.) The other recording (TG12) was performed with both females and RA-implanted males carrying a backpack microphone.
(EPS)

Movie S1 **Zebra finch pair with wireless transmitters.**
(MOV)

Movie S2 **Singing male with electrophysiology transmitter.**
(MOV)

Movie S3 **Aggressive behavior of a male carrying an electrophysiology transmitter.**
(MOV)

Movie S4 **Detailed view of audio transmitter mounted on a female zebra finch.**
(MOV)

## References

[1] Nottebohm F, Stokes TM, Leonard CM (1976) Central Control of Song in Canary, Serinus-Canarius. Journal of Comparative Neurology 165: 457–486. 1262540 10.1002/cne.901650405

[2] Simpson HB, Vicario DS (1990) Brain pathways for learned and unlearned vocalizations differ in zebra finches. J Neurosci 10: 1541–1556. 2332796 10.1523/JNEUROSCI.10-05-01541.1990 PMC6570078

[3] Liu WC, Wada K, Jarvis ED, Nottebohm F (2013) Rudimentary substrates for vocal learning in a suboscine. Nature Communications 4. 10.1038/ncomms3082 23823977

[4] Speirs EAH, Davis LS (1991) Discrimination by Adelie Penguins, Pygoscelis-Adeliae, between the Loud Mutual Calls of Mates, Neighbors and Strangers. Animal Behaviour 41: 937–944.

[5] Poesel A, Dabelsteen T (2006) Three vocalization types in the blue tit Cyanistes caeruleus: a test of the different signal-value hypothesis. Behaviour 143: 1529–1545.

[6] Zann RA (1996) The Zebra Finch; M. PC, editor. Oxford: Oxford University Press.

[7] Beckers GJL, Gahr M (2010) Neural Processing of Short-Term Recurrence in Songbird Vocal Communication. PLoS One 5: -. 10.1371/journal.pone.0011129 PMC2886052 20567499

[8] Morris D (1954) The Reproductive Behaviour of the Zebra Finch (Poephila-Guttata), with Special Reference to Pseudofemale Behaviour and Displacement Activities. Behaviour 6: 271–322.

[9] Amy M, Sprau P, de Goede P, Naguib M (2010) Effects of personality on territory defence in communication networks: a playback experiment with radio-tagged great tits. Proceedings Biological sciences/The Royal Society 277: 3685–3692. 10.1098/rspb.2010.0598 PMC2982238 20591859

[10] Yu AC, Margoliash D (1996) Temporal Hierarchical Control of Singing in Birds. Science 273: 1871–1875. 8791594 10.1126/science.273.5283.1871

[11] Hahnloser RH, Kozhevnikov AA, Fee MS (2002) An ultra-sparse code underlies the generation of neural sequences in a songbird. Nature 419: 65–70. 12214232 10.1038/nature00974

[12] Margoliash D (1997) Functional organization of forebrain pathways for song production and perception. Journal of neurobiology 33: 671–693. 9369466 10.1002/(sici)1097-4695(19971105)33:5<671::aid-neu12>3.0.co;2-c

[13] Long MA, Jin DZZ, Fee MS (2010) Support for a synaptic chain model of neuronal sequence generation. Nature 468: 394–399. 20972420 10.1038/nature09514 PMC2998755

[14] Schregardus DS, Pieneman AW, Ter Maat A, Jansen RF, Brouwer TJF, et al (2006) A lightweight telemetry system for recording neuronal activity in freely behaving small animals. Journal of Neuroscience Methods 155: 62–71. 16490257 10.1016/j.jneumeth.2005.12.028

[15] Marler P (2004) Bird calls - Their potential for behavioral neurobiology. Behavioral Neurobiology of Birdsong 1016: 31–44. 10.1196/annals.1298.034 15313768

[16] Seyfarth RMC, D.L (2010) Production, usage, and comprehension in animal vocalizations. Brain and Language 115: 92–100. 19944456 10.1016/j.bandl.2009.10.003

[17] Miller CT, Beck K, Meade B, Wang XQ (2009) Antiphonal call timing in marmosets is behaviorally significant: interactive playback experiments. Journal of Comparative Physiology a-Neuroethology Sensory Neural and Behavioral Physiology 195: 783–789. 10.1007/s00359-009-0456-1 PMC3787898 19597736

[18] Mathevon N, Vignal C, Mottin S (2008) Mate recognition by female zebra finch: Analysis of individuality in male call and first investigations on female decoding process. Behavioural Processes 77: 191–198. 17980974 10.1016/j.beproc.2007.09.003

[19] Soltis J, Leong K, Savage A (2005) African elephant vocal communication I: antiphonal calling behaviour among affiliated females. Animal Behaviour 70: 579–587.

[20] Silcox AP, Evans SM (1982) Factors Affecting the Formation and Maintenance of Pair Bonds in the Zebra Finch, Taeniopygia-Guttata. Animal Behaviour 30: 1237–1243.

[21] Caryl PG (1976) Sexual-Behavior in Zebra Finch Taeniopygia-Guttata - Response to Familiar and Novel Partners. Animal Behaviour 24: 93–107.

[22] Elie JE, Mariette MM, Soula HA, Griffith SC, Mathevon N, et al (2010) Vocal communication at the nest between mates in wild zebra finches: a private vocal duet? Animal Behaviour 80: 597–605.

[23] Kroodsma DE, Konishi M (1991) A Suboscine Bird (Eastern Phoebe, Sayornis-Phoebe) Develops Normal Song without Auditory-Feedback. Animal Behaviour 42: 477–487.

[24] Gahr M (2007) Sexual Differentiation of the Vocal Control System of Birds. Genetics of Sexual Differentiation and Sexually Dimorphic Behaviors 59: 67–105. 10.1016/S0065-2660(07)59003-6 17888795

[25] Lohmann R, Gahr M (2000) Muscle-dependent and hormone-dependent differentiation of the vocal control premotor nucleus robustus archistriatalis and the motornucleus hypoglossus pars tracheosyringealis of the zebra finch. Journal of Neurobiology 42: 220–231. 10640329 10.1002/(sici)1097-4695(20000205)42:2<220::aid-neu6>3.0.co;2-e

[26] Riebel K, Hall ML, Langmore NE (2005) Female songbirds still struggling to be heard. Trends in Ecology & Evolution 20: 419–420. 16701408 10.1016/j.tree.2005.04.024

[27] Garamszegi LZ, Pavlova DZ, Eens M, Moller AP (2007) The evolution of song in female birds in Europe. Behavioral Ecology 18: 86–96.

[28] Price JJ, Lanyon SM, Omland KE (2009) Losses of female song with changes from tropical to temperate breeding in the New World blackbirds. Proceedings of the Royal Society B-Biological Sciences 276: 1971–1980. 10.1098/rspb.2008.1626 PMC2677260 19324802

[29] Schmidt MF, Konishi M (1998) Gating of auditory responses in the vocal control system of awake songbirds. Nature Neuroscience 1: 513–518. 10196550 10.1038/2232

[30] Fee MS, Leonardo A (2001) Miniature motorized microdrive and commutator system for chronic neural recording in small animals. Journal of Neuroscience Methods 112: 83–94. 11716944 10.1016/s0165-0270(01)00426-5

[31] Tchernichovski O, Nottebohm F, Ho CE, Pesaran B, Mitra PP (2000) A procedure for an automated measurement of song similarity. Animal Behaviour 59: 1167–1176. 10877896 10.1006/anbe.1999.1416

[32] Abeles M (1982) Quantification, smoothing, and confidence limits for single-units’ histograms. J Neurosci Methods 5: 317–325. 6285087 10.1016/0165-0270(82)90002-4

[33] Team RC (2013) R: A language and environment for statistical computing. R Foundation for Statistical Computing, Vienna, Austria.

[34] Hahnloser RH, Kozhevnikov AA, Fee MS (2006) Sleep-related neural activity in a premotor and a basal-ganglia pathway of the songbird. Journal of neurophysiology 96: 794–812. 16495362 10.1152/jn.01064.2005

[35] Spiro JE, Dalva MB, Mooney R (1999) Long-range inhibition within the zebra finch song nucleus RA can coordinate the firing of multiple projection neurons. J Neurophysiol 81: 3007–3020. 10368416 10.1152/jn.1999.81.6.3007

