# Supplementary figures and images for: Zebra Finch Mates Use Their Forebrain Song System in Unlearned Call Communication
Source: PLoS One. 2014 Oct 14;9(10):e109334. doi: 10.1371/journal.pone.0109334 (PMC4196903; doi:10.1371/journal.pone.0109334)

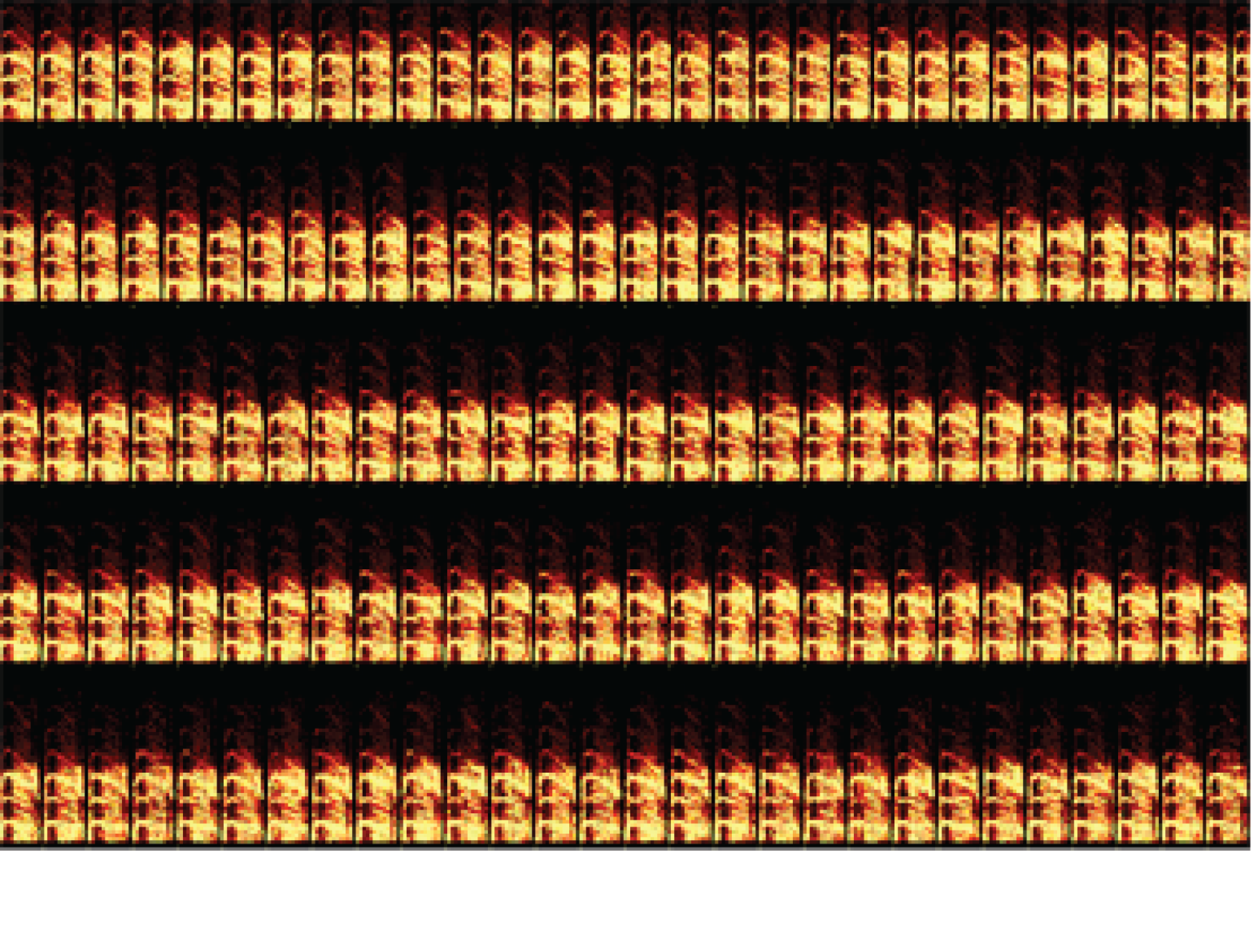

Supplement: Figure S5 — Accuracy of syllable sorting. Example of a song syllable sorted on the basis of mean, modal and fundamental frequencies as well as their standard deviations, duration and wiener entropy and its SD. (TIF) [file pone.0109334.s005.tif]
